# Assessment of knowledge and practice of nurses regarding infection prevention and associated factors at Debre Tabor Comprehensive Specialized Hospital, Northwest Ethiopia

**DOI:** 10.3389/fpubh.2023.1225570

**Published:** 2024-01-08

**Authors:** Tesfahun Zemene Tafere, Tadele Biresaw Belachew, Dejen Getaneh Feleke, Gashaw Mekete Adal

**Affiliations:** ^1^Department of Health Systems and Policy, Institute of Public Health, College of Medicine and Health Sciences, University of Gondar, Gondar, Ethiopia; ^2^Department of Pediatrics and Child Health Nursing, College of Health Sciences, Debre Tabor University, Debre Tabor, Ethiopia; ^3^Department of Nursing and Child Health, Debre Tabor Health Science College, Debre Tabor, Ethiopia

**Keywords:** hospital, infection, knowledge, nurses, practice, risk factors

## Abstract

**Background:**

Healthcare-Acquired Infections have a major negative impact on the global healthcare delivery system, resulting in increased morbidity and mortality and excessive healthcare resource utilization. Infection prevention and control is the main healthcare agenda nationwide. However, it remains a challenge to achieving Sustainable Development Goals regarding healthcare issues. This study aims to assess nurses' knowledge and practice regarding infection prevention and associated factors at Debre Tabor Comprehensive Specialized Hospital, Northwest Ethiopia.

**Methods:**

An institutional-based cross-sectional study was carried out from May 1 2021 to June 30 2021 at Debre Tabor Comprehensive Specialized Hospital, Ethiopia. A total of 219 nurses participated in this study. A stratified random sampling technique with a pre-tested structured self-administered questionnaire was used to collect data. A Multivariable logistic regression model was fitted to identify factors associated with the knowledge and practice of nurses regarding infection prevention.

**Result:**

The current study shows that the overall knowledge and practice of nurses regarding infection prevention are 59.4% (95% CI: 53.0–65.8) and 53% (95% CI: 46.6–59.4), respectively. Educational level of diploma [AOR: 0.8, 95% CI: 0.67–0.83], years of work experience ≤ 10 [AOR: 1.7, 95% CI: 1.3–9.28], and being trained in infection prevention [AOR: 2.5 (2.3–8.0)] were found to be factors that affect the knowledge of nurses about infection prevention. Moreover, ≤ 10 years of work experience [AOR = 1.5, 95% CI: 1.2–10.1] and being trained in infection prevention [AOR = 2.2, 95% CI: 1.94–13.5] were found to be factors that affect the practice of nurses regarding infection prevention.

**Conclusions and recommendations:**

Most nurses were knowledgeable and had good practices. However, a substantial proportion of nurses had inadequate knowledge and practice regarding infection prevention. The nurses' knowledge and practice were associated with years of work experience and status of training on infection prevention. Moreover, the education level of nurses was another predictor variable of the knowledge of nurses. Therefore, healthcare workers in the hospital setting should adhere to the national infection prevention protocols. The policy designers should provide training for nurses on infection prevention to improve knowledge and practice in this area. Furthermore, to attain more detailed information, future research should involve a qualitative study.

## Background

Infection is defined as the colonization of body tissue by bacteria and other microorganisms (1). Due to the failure to prevent infection globally, more than 400 million individuals worldwide have chronic hepatitis B virus (HBV) infection, and up to one million people may pass away from an HBV-related illness each year (2). In healthcare environments, infections can spread from Health Care Providers (HCPs) to patients, patients to HCPs, patients to patients, and HCP to HCP. Healthcare-Acquired Infections (HAI) are a major problem in the world within the healthcare delivery system (3).

According to the global estimation of the global burden of disease attributable to contaminated sharps injuries among healthcare professionals, HCWs may have contracted 16,000 HCV, 66,000 HBV, and 1,000 HIV infections worldwide in 2000 as a result of their job-related exposure to percutaneous wounds (4). The general HCAI predisposing factors are associated with characteristics of the patient, such as age, underlying disease, comorbidities, and reduced host defenses.

Infection prevention and control (IPC) is a vital, continuing requirement for safeguarding patients and healthcare workers (HCWs) against the transmission of infectious diseases in healthcare settings (5). Globally, the prevalence of HCV among 100 countries was 1%, with viremia infections in 71.1 million people (6).

An elaborative global health system has been established as a defense against both known and unknown infectious disease threats in order to slow the spread of infectious diseases. The system is made up of several formal and informal networks of organizations that work with different stakeholders; have different objectives, methods, and levels of accountability; operate at various regional scales (e.g., local, national, regional, or global); and span the public, for-profit, and non-profit sectors (1).

Existing studies have tried to assess the knowledge and practice of healthcare workers regarding infection prevention and associated factors (7–9); however, the knowledge and practice of nurses in particular, who carry out the highest burden of healthcare activities, regarding infection prevention and associated factors are not well addressed. Furthermore, less is known about the burden associated with substandard care and ineffective infection control in settings with limited resources (10–12). Therefore, this study aims to assess nurses' knowledge and practice regarding infection prevention and associated factors at Debre Tabor Comprehensive Specialized Hospital, Northwest Ethiopia.

## Method and materials

### Study design and setting

The institutional-based cross-sectional study design was carried out from May 1 2021 to June 30 2021 at Debre Tabor Comprehensive Specialized Hospital Ethiopia. According to the information obtained from the Debre Tabor city administrative health department, the hospital served as a general hospital until the end of 2019 and upgraded to a Comprehensive Specialized Hospital in 2020. The hospital is organized into surgical, medical, gynecology, pediatrics, ophthalmology, emergency, and intensive care unit wards. Currently, it has a total of 125 inpatient beds in all wards and 534 staff providing health care services for a population of ~2,651,350.

All nurses working in Debre Tabor Comprehensive Specialized Hospital, providing healthcare services, and who had at least one of four possible contacts (patients, medical equipment, linens, and high-risk wastes) were eligible to be included in the study. The study excluded nurses who were on annual or maternity leave at the time the data were collected as well as those who were ill and unable to respond to the questions.

### Population

All nurses working in Debre Tabor Comprehensive Specialized Hospital were the source population, while nurses working in Debre Tabor Comprehensive Specialized Hospital and providing healthcare services who had at least one of four possible contacts (patient, medical equipment, linens, and high-risk waste) were the study population.

### Sample size and sampling procedure

The sample size was determined using the single population proportion formula. A previous study on the level of knowledge and practices of nurses regarding infection prevention higher proportion was considered and demonstrated that the proportion of nurses with good knowledge regarding infection prevention was 84.7% (8), with a 95% confidence level, 5% margin of error, and 10% non-response rate, which yields the final sample size of 219. A stratified random sampling technique was used to select the required sample size of 219 nurses. Hospital departments were classified into six main strata with nearly the same working conditions: (1) Medical, (2) Surgical, (3) Pediatrics, (4) Gynecology, (5) Ophthalmology, and (6) Emergency and Intensive Care departments. The proportional allocation was taken from each stratum and then a simple random sampling technique was applied.

### Variables and measurements

The dependent variables studied were the knowledge and practice of nurses regarding infection prevention. The independent variables were sex, job category, educational level, year of work experience, working hours, work shift, status of training, marital status, monthly income, duration status, and level of hospital previously worked in and currently working in.

Nurses' knowledge of infection prevention was assessed by 18 “yes or no” questions. A scoring system was utilized in which the respondents' correct and incorrect answers provided for the questions were allocated “1 and 0” points, respectively. Knowledge scores were summed up to give a total knowledge score for each respondent. There were two types of responses based on the total score for the knowledge questions, which ranged from 0 to 18. Respondents who scored the mean and above were knowledgeable and those who scored below the mean were not knowledgeable (13, 14).

The nurses' practice regarding infection prevention was measured by 15 items to which the responses were “yes” or “no.” Similar methods were used to analyze the practice: a score of 1 was given for good practice and a score of 0 for poor practice. Hence, the range of the overall score for infection prevention practice was from 0 to 15. Respondents who scored mean and above were classified as good practice and those who scored below the mean were classified as poor practice (13, 14).

### Operational definitions

- Knowledge: Nurses' understanding of infection prevention.
- Knowledgeable: Knowledge status of nurses when they scored 50% and above on the knowledge questions.
- Not knowledgeable: Nurses' status of knowledge when they scored <50% on the knowledge questions.
- Practice: The act of performing a given procedure(s) in accordance with a predetermined standard.
- Good practice: Practice level of nurses when they scored 50% and above on the practice questions.
- Poor practice: Practice level of nurses when they scored <50% on the practice questions (13, 14).

### Data collection tools and procedures

The data collection tool was a three-part questionnaire involving socio-demographic variables, knowledge, and practice-related variables on infection prevention and control. Data were gathered using a pre-tested, structured self-administered questionnaire. The data collection tool was developed by reviewing relevant literature (13, 15, 16) and by adapting the content from related studies (13, 14). The instrument for collecting data was initially written in English; it was then translated into Amharic (the local tongue) and, finally, back into English to ensure uniformity. The data collection process was conducted by four data collectors and two supervisors.

### Data quality assurance and control

A day of training focused on the objectives of the study, how to get consent, maintain confidentiality, and basic data collection techniques was provided for both data collectors and supervisors.

The instruments were undergone preliminary testing to enhance reliability on 5% (11) of individuals in the total sample with traits that the target population shares and the necessary amendment was done accordingly. Questionnaires were checked for consistency and completeness. The data collection process was checked by supervisors daily and appropriate action was taken according to the findings. Alpha Cronbach values of 0.78 and 0.822 demonstrated the internal consistency of items for the knowledge and practice sections, respectively.

### Data processing and analysis

Data were entered into Epidata version 4.6.0 software and STATA version 13 statistical software for analysis. The analysis was both descriptive and analytical. The descriptive analysis was presented using texts, frequency, and cross-tabulation.

A binary logistic regression model was fitted to assess the association between the dependent and independent variables, and those variables with a *p* < 0.2 were entered into a multivariable logistic regression to control the confounding effect and to estimate the independent variables of knowledge and practice regarding infection prevention. A *p* < 0.05 at a 95% confidence interval was considered statistically significant. The appropriate model goodness-of-fit was checked by the Hosmer Lemeshow test which was 91.8%, indicating that the model fitness was good. Finally, significant findings in the multivariable analysis were interpreted.

## Result

### Socio-demographic characteristics of nurses

A total of 219 nurses were enrolled in this study. The mean (SD) age of the nurses was 36.02 ± 14.56. The monthly income and duration of training were 6642.2 ± 2279.29 and 4.2 ± 4.3, respectively. The majority, 56.2% (123), were female, 37.9% belonged to the age group 41+ years, 81.7% had a bachelor's degree, and 59.4% were staff nurses by profession. Moreover, 58% and 70.3% of the nurses were married and earned a monthly salary of 73.3 US$-146.5 US$ respectively. Approximately one-third (29.7%) and three-quarters (75.8%) of participants had work experience of > 10 years and were working on day shifts, respectively (Table 1).

**Table 1:** Socio-demographic characteristics of nurses at Debre Tabor Comprehensive Specialized Hospital, Northwest Ethiopia, 2021 (*n* = 219).

**Variables**	**Frequency**	**Percentage (%)**
**Age (in years)**		
20–25	24	11
26–30	52	23.7
31–40	60	27.4
41+	83	37.9
**Sex**		
Male	96	43.8
Female	123	56.2
**Religion**		
Orthodox Christian	179	81.7
Protestant	16	7.3
Muslim	24	11
**Educational level**		
Diploma	29	13.2
Bachelor	178	81.3
Masters and above	12	5.5
**Job category**		
Supervisor nurse	27	12.3
In charge nurse	62	28.3
Staff nurse	130	59.4
**Marital status**		
Never married	127	58
Married	70	32
Divorced	22	10
**Monthly income**		
73.3–146.5 (US$)	154	70.3
146.5+ (US$)	65	29.7
**Work experience (in years)**		
≤ 10	154	70.3
>10	65	29.7
**Work shift**		
Day	166	75.8
Night	53	24.2
**Trained in infection prevention**		
Yes	27	12.3
No	192	87.7
**Duration of training (in days)**		
1-5	16	7.3
>5	11	5
**Currently working in**		
OPD	73	33.3
Triage	14	6.4
Ward	132	60.3

### Knowledge of the nurses regarding infection prevention

The study revealed that the overall knowledge of nurses about infection prevention was 59.4% (95% CI: 53.0–65.8). Approximately 82.6% of nurses knew about safety precautions for infection prevention and 87.7% were aware of the recommended guidelines for hand hygiene. Similarly, 86.8% and 13.2% of the study participants were aware of the IPC team and had undertaken infection prevention-related training in the past 12 months.

Moreover, 83.6% and 65.8% of nurses were aware of the availability of infection prevention guidelines in their working area and the availability of personal protective equipment (PPE) at all times, respectively. Likewise, 86.6%, 14.2%, and 18.3% of nurses were aware that disinfection prevents hospital-acquired infection, used chemical sterilization techniques for all equipment, and employed physical sterilization (heat and radiation) techniques for all equipment used, respectively. Similarly, 77.2%, 90.0%, and 85.8% of the study participants believed that all microorganisms, including spores, are destroyed by autoclaving, all equipment must be decontaminated before sterilization, and protective clothing minimizes hospital-acquired infection, respectively (Table 2).

**Table 2:** Knowledge of the nurses regarding infection prevention at Debre Tabor Comprehensive Specialized Hospital, Northwest Ethiopia, 2021 (*n* = 219).

**Variables**	**Frequency**	**Percentage (%)**
**Knowing about safety precautions**		
Yes	181	82.6
No	38	17.4
**Presence IPC team**		
Yes	190	86.8
No	29	13.2
**Trained in infection prevention in the past 12 months**		
Yes	29	13.2
No	190	86.8
**Availability of infection prevention guidelines in the work area**		
Yes	183	83.6
No	36	16.4
**Availability of PPE at all times**		
Yes	144	65.8
No	75	34.2
**Availability of water for 24 h**		
Yes	106	48.4
No	113	51.6
**Knowing the impact of HCAIs on clinical outcomes**		
Yes	184	84
No	35	16
**Knowing disinfection prevents hospital-acquired infection**		
Yes	194	88.6
No	25	11.4
**Chemical sterilization technique used for all equipment**		
Yes	31	14.2
No	188	85.8
**Believe that all microorganisms including spores are destroyed**		
**by autoclaving**		
Yes	169	77.2
No	50	22.8
**All equipment needs decontamination before sterilization**		
Yes	199	90.9
No	20	9.1
**Protective clothing minimizes hospital-acquired infection**		
Yes	188	85.8
No	31	14.2
**Proper handling of working equipment decreases the risk**		
**of contamination**		
Yes	202	92.2
No	17	7.8
**Vaccinated for HBV**		
Yes	19	8.7
No	200	91.3
**Presence of a system for reporting accidental exposure to**		
**blood and body fluids**		
Yes	179	81.7
No	40	18.3
**Know to take PEP while exposed to body fluid or needle-stick**		
**injuries of HIV-infected patients**		
Yes	193	88.1
No	26	11.9

### The practice of the nurses regarding infection prevention

In this study, 53% (95% CI: 46.6–59.4) of nurses were found to have good practice. Moreover, approximately three-quarters (76.7%) of nurses responded that they wash their hands with soap and water after taking a sample and 182 (83.1%) responded that they wash their hands immediately after contact with body fluids and contaminated items.

Furthermore, 166 (75.8 %), 206 (94.1%), and 182 (83.2%) nurses reported that they discard sharp materials in a safety box, wear goggles to protect their eyes during procedures, and wear a mask during sputum sample collection and processing, respectively.

On the contrary, 84 (38.4 %) and 26 (11.9%) of nurses reported that they recap needles before disposal and do not wear gowns properly for every procedure, respectively. Wearing an apron, covering wounds and cuts on the skin before starting work, being vaccinated against common pathogens, and eating or drinking in the work area were 48.4%, 68.5%, 17.8%, and 48.8%, respectively.

However, 49 (22.4%), 168 (76.7%), and 180 (82.2%) of the study participants responded that they had been exposed to needle-stick injury, soak contaminated medical equipment in 0.5% chlorine solution, and separate wastes infectious and noninfectious, respectively (Table 3).

**Table 3:** Practice of the nurses regarding infection prevention at Debre Tabor Comprehensive Specialized Hospital, Northwest Ethiopia, 2021 (*n* = 219).

**Variables**	**Frequency**	**Percentage (%)**
**Washing hands with soap and water after taking a sample**		
Yes	168	76.7
No	51	23.3
**Washing hands immediately after coming into contact with**		
**blood, body fluids, or contaminated items**		
Yes	182	83.1
No	37	16.9
**Discard sharp materials in a safety box**		
Yes	166	75.8
No	53	24.2
**Discard needles in the sharps bin**		
Yes	168	76.7
No	51	23.3
**Wear goggles to protect your eyes during procedures that**		
**generate a spray of blood or body fluids**		
Yes	206	94.1
No	13	5.9
**Wear a mask during sputum sample collection and processing**		
Yes	182	83.1
No	37	16.9
**Recap needles before disposal**		
Yes	84	38.4
No	135	61.6
**Wear a gown properly for every procedure**		
Yes	193	88.1
No	26	11.9
**Wear an apron when blood or body fluid splash is expected**		
Yes	106	48.4
No	113	51.6
**Cover wounds and cuts on the skin before starting work**		
Yes	150	68.5
No	69	31.5
**Vaccinated for common pathogens**		
Yes	39	17.8
No	180	82.2
**Eat or drink in your work area**		
Yes	85	38.8
No	134	61.2
**Exposure to needle-stick injury**		
Yes	49	22.4
No	170	77.6
**Soak contaminated medical equipment in 0.5% chlorine solution**		
For 10 min	168	76.7
For 1 h	39	17.8
For 24 h	12	5.5
**Separation of waste into infectious and non-infectious**		
Yes	180	82.2
No	39	17.8

### Factors associated with knowledge of nurses about infection prevention

In the bivariable analysis, age, sex, educational level, years of work experience, work shift, and ever having been trained on infection prevention were the predictor variables associated with the knowledge of nurses about infection prevention.

However, only education level, years of work experience, and status of training on infection prevention were factors associated with the knowledge of nurses about infection prevention in the multivariable logistics regression analysis.

Nurses with an educational level of diploma were 20% less knowledgeable than nurses with a master's or above [AOR = 0.8, 95% CI: 1.7–8.3, *P* = 0.04]. Moreover, nurses with ≤ 10 years of work experience were 1.7 times more likely to be knowledgeable compared to nurses with >10 years of work experience [AOR = 1.7, 95% CI: 1.3–9.3, *P* = 0.002]. Similarly, nurses who had ever been trained on infection prevention were 2.5 times more knowledgeable than those who had never [AOR = 2.5, 95% CI: 2.3–8.0, *P* = 0.03] (Table 4).

**Table 4:** Factors associated with knowledge of the nurses regarding infection prevention at Debre Tabor Comprehensive Specialized Hospital, Northwest Ethiopia, 2021 (*n* = 219).

**Variables**	**Knowledge of infection prevention**		**COR (95% CI)**	**AOR (95% CI)**	***P*-value**
	**Yes**	**No**			
**Age (in years)**					
20–25	20	4	4.37 (1.24–9.53)^*^	4.0 (0.86–9.35) 1.6 (0.9–6.0)	0.3
26–30	3	22	1.5 (0.6–8.0)	1.8 (0.9–6.0)	1.08
31–40	50	10	1.65 (0.7–10.0)	1	0.5
41+	43	40	1		
**Sex**					
Male	69	58	2.3 (1.56–3.99)^*^	2.2 (0.52–7.87)	1.4
Female	54	151	1	1	
**Educational level**					
Diploma	20	9	0.56 (0.6–0.93)^*^	0.8 (0.67–0.83)^**^	0.04
BSc	96	82	0.72 (0.77–14.0)	0.8 (0.75–12.0)	0.9
MSc and above	10	2	1	1	
**Work experience (in years)**					
≤ 10	36	54	1.89 (1.8–12.0)^*^	1.7 (1.3–9.28)^**^	0.002
>10	69	60	1	1	
**Work shift**					
Day shift	100	54	0.67 (0.7–0.89)^*^	0.9 (0.09–11.0)	1.6
Night shift	16	49	1	1	
**Trained in infection prevention**					
Yes	100	66	2.1 (1.5–10.3)^*^	2.5 (2.3–8.0)^**^	0.03
No	16	37	1	1	

AOR, Adjusted Odds Ratio; COR, Crude Odds Ratio; CI, Confidence Interval, ^*^p < 0.2, ^**^p < 0.05.

### Factors associated with the practice of the nurses regarding infection prevention

In the bivariable logistics regression analysis, sex, education level, years of work experience, work shift, and status of training on infection prevention were factors that were significantly associated with nurses' practice regarding infection prevention, whereas only years of work experience and status of training on infection prevention were significantly associated with the infection prevention practice of nurses in the multivariable analysis.

Nurses with work experience of ≤ 10 years had 1.5 good than nurses who had a work experience of >10 years [AOR = 1.5, 95% CI: 1.2–10.1, *P* = 0.003]. Moreover, nurses who had been trained on infection prevention had 2.2 times better practice than those who had not [AOR = 2.2, 95% CI: 1.94–13.5, *P* = 0.04] (Table 5).

**Table 5:** Factors associated with the practice of the nurses regarding infection prevention at Debre Tabor Comprehensive Specialized Hospital, Northwest Ethiopia, 2021 (*n* = 219).

**Variables**	**Practice regarding infection prevention**		**COR (95% CI)**	**AOR (95% CI)**	***P*-Value**
	**Yes**	**No**			
**Sex**					
Male	69	58	1.22 (1.56–3.99)^*^	1.0 (0.2–1.57)	1.3.
Female	54	151	1	1	
**Educational level**					
Diploma	20	9	1.6 (1.3–1.92)^*^	0.9 (0.73–6.34)	1.9.
BSc	96	82	0.72 (0.77–14.0)	0.6 (0.5–11.0)	2.2.
MSc and above	10	2	1	1	
**Work experience (in years)**					
<10	100	54	1.57 (1.4–15.00)^*^	1.5 (1.2–10.1)^**^	0.003
>10	69	60	1	1	
**Work shift**					
Day shift	100	167	0.5 (0.3–0.69)^*^	0.7 (0.2–2.3)	3.4.
Night shift	16	49	1	1	
**Trained in infection prevention**					
Yes	100	66	1.6 (1.5–12.1)^*^	2.2 (1.94–13.5)^**^	0.04
No	16	37	1	1	

AOR, Adjusted Odds Ratio; COR, Crude Odds Ratio; CI, Confidence Interval, ^*^p < 0.2, ^**^p < 0.05.

## Discussion

This study revealed that 59.4% (95% CI: 53.0–65.8) of nurses were knowledgeable about infection prevention. In this study, it is indicated that a substantial proportion of respondents (40.6%) in the hospital had inadequate knowledge about infection prevention, whereas 53% (95% CI: 46.6–59.4) of nurses had good practice in infection prevention.

The knowledge of nurses about infection prevention in the current study was higher than similar studies conducted in Amhara regional state referral hospitals, Addis Ababa, and Eastern Ethiopia, which reported that 40.7%, 46.3%, and 47,7% of the nurses were knowledgeable, respectively (17–19), but lower compared with a similar study conducted in Wolaita Sodo, Afar, Debre Markos, and Bahir Dar, where the overall knowledge of nurses about infection prevention was 99.3%, 65.9%, 84.7%, and 74.5%, respectively (8, 20–22). This difference might be due to differences in the provision of training provided for nurses and the previous experience of nurses.

Another possible justification for this di?erence might be the differences in socioeconomic status, study setting, and sample size and the use of different data collection tools.

However, this finding is similar to studies conducted in Dubti referral hospitals and Zambia, where the overall knowledge of nurses was reported to be 48.4% and 48.88%, respectively (23, 24). A possible reason for the similarity between the current study, Dubti referral hospital, and Zambia might be the use of a similar study design (cross-sectional) and study population (nurses).

Moreover, in this study, the practice of nurses regarding infection prevention was higher compared with the Gamo Gofa zone, Ethiopia, which reported that the overall practice of nurses regarding infection prevention was 39.9% (25), but lower compared with studies conducted in Addis Ababa, Dessie, Nigeria, Yemen, and Saudi Arabia, where the practice of nurses regarding infection prevention was reported to be 66.1%, 77%, 77.9%, 71%, and 92.1%, respectively (26–29). Possible reasons for this discrepancy might be the time gap and the improper supply of PPE, including disinfectants, in the study areas.

The current study also assessed factors that affect the knowledge and practices of nurses regarding infection prevention. The findings indicate that the knowledge of nurses about infection prevention was associated with education level, years of work experience, and status of training on infection prevention. Furthermore, nurses with an education level of diploma were less knowledgeable than nurses with an educational level of master's or above. This study was supported by the study conducted in Debre Markos referral hospital, Northwest Ethiopia, which showed that healthcare workers with an educational level of BSC or above were two times more likely to be knowledgeable than those with Diplomas (8). This could be attributed to the possibility that more educated nurses have better access to knowledge about infection prevention.

In this study, it is shown that years of work experience and knowledge of nurses had an indirect association. Nurses with fewer years of work experience were more likely to be knowledgeable compared to nurses who had more years of work experience. This might be due to the recalling effect, meaning that nurses with fewer years of work experience might recall courses taken during their education more easily than nurses with more years of work experience.

Moreover, nurses who had ever been trained on infection prevention were more knowledgeable than nurses who had never. This finding is consistent with the study conducted in Debre Markos referral hospital, Northwest Ethiopia, where healthcare professionals who hadn't received infection prevention training were 75% less likely to be knowledgeable about infection prevention than those who had received training on infection prevention (8). This consistency might be due to the similarity of the health facility setup. This implies that training increases the chances of nurses accessing up-to-date information about infection prevention.

This study also showed that years of work experience and status of training on infection prevention were significantly associated with the infection prevention practice of nurses.

Nurses with fewer years of work experience were more likely to have good practice compared to nurses who had more years of work experience. This might be because nurses with more years of work experience may be bored, leading to negligence in the practice of nurses regarding the recommended protocols. This finding contrasts with the study done in Bahir Dar City, Ethiopia, where HCWs with work experience of 10 years and above were four times more likely to practice infection prevention than those with less than 10 years of experience (14). This difference might be due to the differences in the characteristics of the health institutions where the studies were conducted.

Similarly, nurses who had been trained on infection prevention had good practice compared to those who had not. This might be training enhanced the standards of practice of nurses. This implies that providing continuous training for nurses regarding infection prevention has an impact on putting their knowledge into practice.

## Limitations of the study

Despite extensive efforts to minimize limitations, the study has some limitations that should be acknowledged. The study was conducted at Debre Tabor Comprehensive Specialized Hospital and, therefore, might not be representative of all health professionals across Ethiopia. There might be a recall bias during data collection time that could affect the true level of practice and knowledge of nurses regarding infection prevention. The other possible limitation of the study is that it only quantitatively assessed infection prevention, not qualitatively.

The strength of the study is that it incorporated two outcome variables (knowledge and practice) to attain more accurate evidence of IPC.

## Conclusion and recommendations

The majority of nurses were knowledgeable and had good practice. However, a substantial proportion of respondents in the hospital had inadequate knowledge and practice regarding infection prevention. Moreover, the nurses' knowledge and practice were associated with years of work experience and status of training on infection prevention. Furthermore, the education level of nurses was another predictor variable of the knowledge of nurses.

Based on the findings of this study, it is recommended that healthcare workers in the hospital adhere to the national infection prevention protocol guidelines. policy makers should educate and train nurses on infection prevention and provide continuous professional development to maintain or improve knowledge and practice regarding infection prevention among nurses. Moreover, to attain more detailed information, future research should include a qualitative study.

## Data availability statement

The raw data supporting the conclusions of this article will be made available by the authors, without undue reservation.

## Ethics statement

Ethical clearance was obtained from the Research Ethics Committee of Debre Tabor University Department of Nursing (Ref. no. DT /12596/214). Written informed consent was obtained from each participant following a brief explanation of the research objectives and data collection process of the study. Participants were also informed about their right to withdraw at any time or to skip a single question or several questions. All methods were carried out in accordance with the Declaration of Helsinki.

## Author contributions

TT conceptualized the study. TT, DF, and TB contributed to the data extraction and analysis. TT, TB, and GA wrote the result interpretation. TT and TB prepared the first draft. TT and DF contributed to the conceptualization, interpretation of results, and substantial revision. TT, TB, DF, and GA revised and finalized the final draft manuscript. All the authors read and approved the final version of the manuscript.
